# Supplementary material for: Leveraging AI to automate detection and quantification of extrachromosomal DNA to decode drug responses
Source: Front Pharmacol. 2025 Feb 3;15:1516621. doi: 10.3389/fphar.2024.1516621 (PMC11830698; doi:10.3389/fphar.2024.1516621)
Supplement: Supplementary file 1 [file DataSheet1.pdf]

Leveraging AI to Automate Detection and Quantification of Extrachromosomal DNA (ecDNA) to Decode Drug Responses

Kohen Goble[c,*], Aarav Mehta[a,*], Damien Guilbaud[d], Jacob Fessler[a], Jingting Chen[b], William Nenad[f,g], Oliver Cope[d], Darby Cheng[c], William Dennis[d], Nithya Gurumurthy[d], Yue Wang[e], Kriti Shukla[c], Christina Ford[d], Elizabeth Brunk[*,c,d,e,f,h,†]

[a] Department of Computer Science, University of North Carolina at Chapel Hill, Chapel Hill, NC 27516

[b] Department of Biochemistry and Biophysics, University of North Carolina at Chapel Hill, Chapel Hill, NC 27516

[c] Department of Chemistry, University of North Carolina at Chapel Hill, Chapel Hill, NC 27516

[d] Integrative Program for Biological and Genome Sciences (IBGS), University of North Carolina at Chapel Hill

[e] Department of Pharmacology, University of North Carolina at Chapel Hill, Chapel Hill, NC 27516

[f] Computational Medicine Program, University of North Carolina at Chapel Hill, Chapel Hill, NC 27516

[g] Curriculum in Bioinformatics and Computational Biology, University of North Carolina at Chapel Hill, Chapel Hill, NC 27516

[h] Lineberger Comprehensive Cancer Center, University of North Carolina at Chapel Hill, Chapel Hill, NC 27516

† Correspondence should be addressed to: Elizabeth Brunk (elizabeth_brunk@med.unc.edu); *These authors contributed equally

Keywords

Cytogenetics, Extrachromosomal DNA, ecDNA, double minute chromosomes, machine learning, Computer Vision, Fluorescence in situ Hybridization, Deep Neural Networks, HSR, drug response, JQ1

Supplementary Information

Supplementary Methods

Automatic Identification of FISH Probes

To automate the identification of FISH probes, we developed a Python script to analyze the RGB content of ground truth ecDNA pixel locations. The script begins by loading the coordinates of all pixels associated with each ecDNA object in the ground truth data. Using these coordinates, the script identifies the same pixel locations in the corresponding RGB image. The red and green content of each pixel is then compared to preset thresholds for each color channel. If any pixel crosses both the red and green thresholds, the ecDNA is counted as containing both the MYC and ERBB2 probes. If only the green threshold is crossed, the ecDNA is counted as having only the MYC probe, and if only the red threshold is crossed, it is counted as having only the ERBB2 probe. This process is repeated for all ecDNA objects in the ground truth data, generating automated counts for ecDNAs containing our probes of interest. In the future, we plan to extend this method to analyze predicted ecDNA locations rather than relying on ground truth data. For the purposes of this study, we demonstrate that the RGB content of the pixels, along with constant thresholds, can accurately identify probes.

To set the red and green thresholds, we began by selecting a random image from the NCIH2170 control dataset and iterated through a large list of preset threshold combinations. For each combination, we calculated the probe counts for three categories: MYC-only, ERBB2-only, and MYC+ERBB2. We then compared these counts to the ground truth data, summing the total differences for each combination. The combination with the smallest total difference was selected. Next, we randomly sampled five additional images from the NCIH2170 control dataset and tested a narrower range of threshold combinations, centered around the previously obtained optimal threshold. The combination that produced the smallest differences across all five images was selected as the final threshold set.

However, this final threshold combination was found to be the smallest in the narrowed list. This bias likely arose due to two main factors. First, most ecDNAs in the dataset contained both MYC and ERBB2 probes, and our ground truth annotations do not perfectly align with the actual ecDNA. Specifically, the ground truth is represented by a single pixel near the center of each ecDNA, which is programmatically expanded into a 5x5 diamond. This expansion inevitably misses some ecDNA pixels, particularly for ecDNA containing both probes, leading to undercounts. Second, the first image used for threshold testing may have contained more ecDNA with only one probe than the rest of the dataset, influencing the initial threshold selection. This caused the final thresholds to be biased towards detecting ecDNA with both probes, resulting in thresholds from the lower end of the refined range.

As a result, our algorithm tends to overcount ecDNA containing only MYC or only ERBB2 probes due to the lower thresholds, while it consistently undercounts ecDNA containing both MYC and ERBB2, as our ground truth data likely misses some relevant pixels. In future work, we aim to select subsets of ecDNAs from each probe category and have annotators confirm the presence of probes. By manually extracting the associated pixels from the RGB images, we will be able to set thresholds that are more accurate and generalizable across different datasets, reducing the bias introduced by imperfect ground truth data and the imbalanced ratio of ecDNA categories.

Supplementary Figures

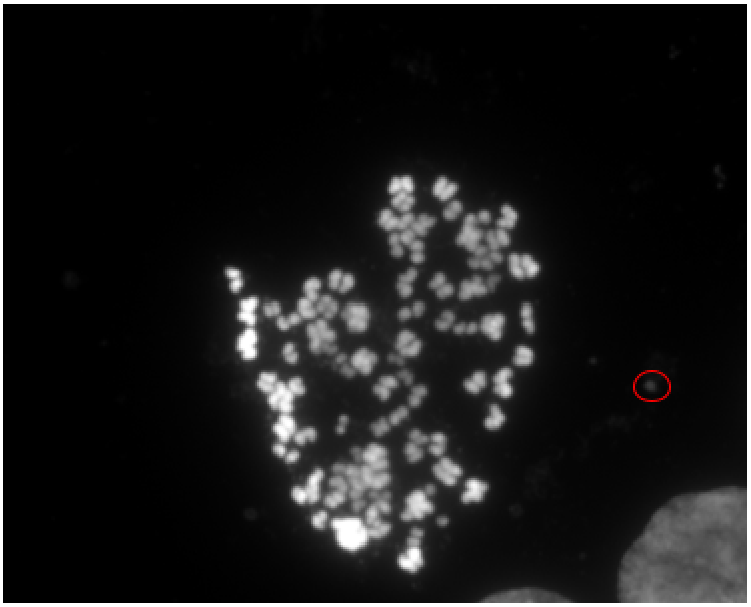

SI Figure 1 | Example of Debris in Metaphase Spread Image. This artifact in the red circle picked up by the DAPI stain is not central to the metaphase spread and likely too large to be ecDNA. This would be considered debris, and would not be counted or included as part of the ROI.

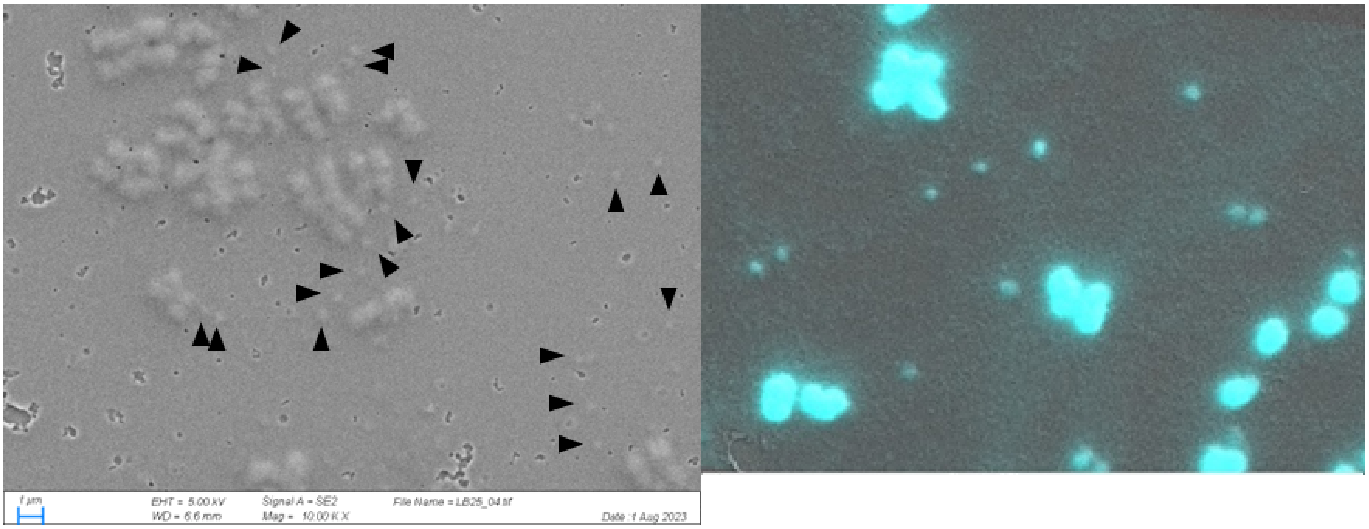

SI Figure 2 | Size of ecDNA from Scanning Electron Microscopy and Correlative Light Electron Microscopy. Figure adapted from Madren, Chen et al. (Biotechniques, 2024). The left panel shows Scanning Electron Microscopy (SEM) images acquired under Os UA EtOH Air conditions, while the right panel presents an image obtained through Correlative Light Electron Microscopy (CLEM). CLEM combines the high resolution of SEM with fluorescence microscopy to confirm the identity of the ecDNA as DNA. In this case, the ecDNA is estimated to be approximately 0.25 µm in size.

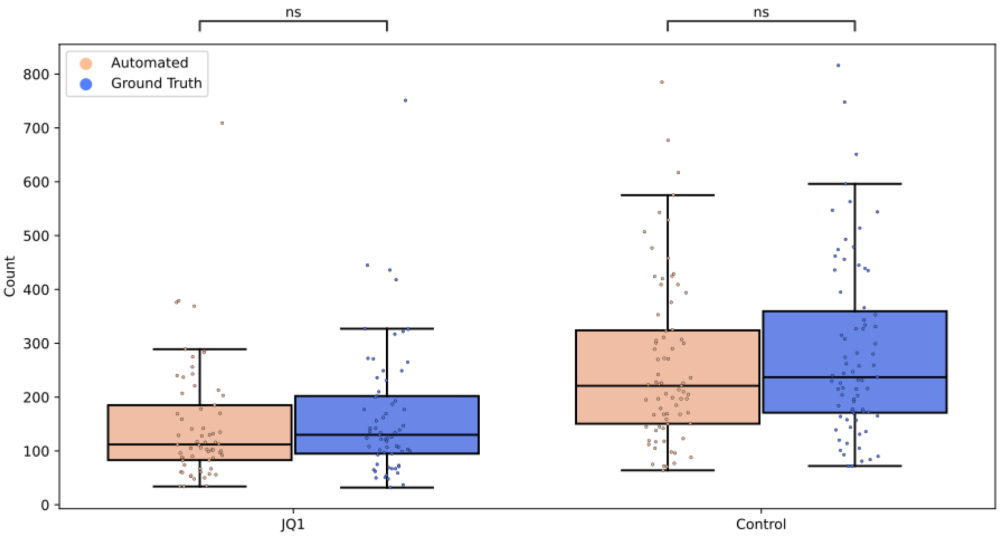

SI Figure 3 | Automated Double MYC and ERBB2 Probe Counting. This figure illustrates the results of our automated probe counting script for identifying ecDNAs with **both MYC and ERBB2** probes in two subsets of the NCIH2170 dataset: JQ1-treated and control cells. In both subsets, the algorithm consistently undercounted the number of ecDNAs containing both probes. For the JQ1-treated subset, the predictions were not significantly different from the ground truth (p = 5.002e-01). Similarly, in the control subset, the predictions were slightly more accurate but still showed no significant difference from the ground truth (p = 6.203e-01). The statistical test used was the Mann-Whitney-Wilcoxon test (two-sided) with a Bonferroni correction applied.

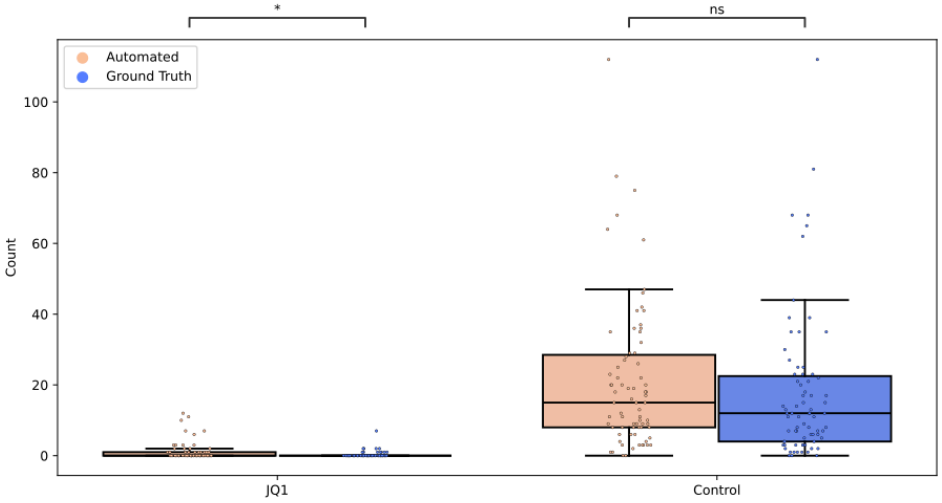

SI Figure 4 | Automated Single ERBB2 Probe Counting. Results of our automated probe counting script for **ERBB2** probe identification in two subsets of the NCIH2170 dataset: JQ1-treated and control cells. In both subsets, the algorithm overestimated the number of ecDNAs with only the ERBB2 probe. For the JQ1-treated subset, predictions were marginally significantly different from the ground truth (p = 1.389e-02). However, this significance may be exaggerated by the small number of ecDNAs with only ERBB2 in this subset—missing even one or two ecDNAs could create a misleading result, despite the algorithm's overall accuracy. In the control subset, the predictions were closer to the ground truth, showing no significant difference (p = 1.898e-01). Statistical analysis was performed using the Mann-Whitney-Wilcoxon test (two-sided) with a Bonferroni correction.

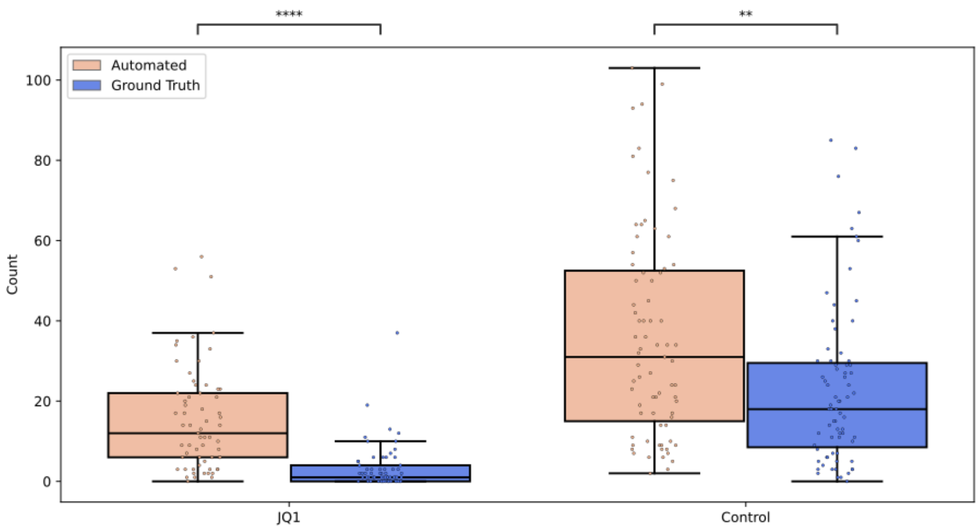

SI Figure 5 | Automated Single MYC Probe Counting. This figure displays the results of our automated probe counting script for MYC probe identification in two subsets of the NCIH2170 dataset: JQ1-treated and control cells. In both subsets, the algorithm overestimated the number of ecDNAs with only the MYC probe. For the JQ1-treated subset, the predictions were significantly different from the ground truth (p = 2.762e-12). In the control subset, the predictions were closer to the ground truth but still showed a significant difference (p = 2.144e-03). The Mann-Whitney-Wilcoxon test (two-sided) was used for statistical analysis, with a Bonferroni correction applied.

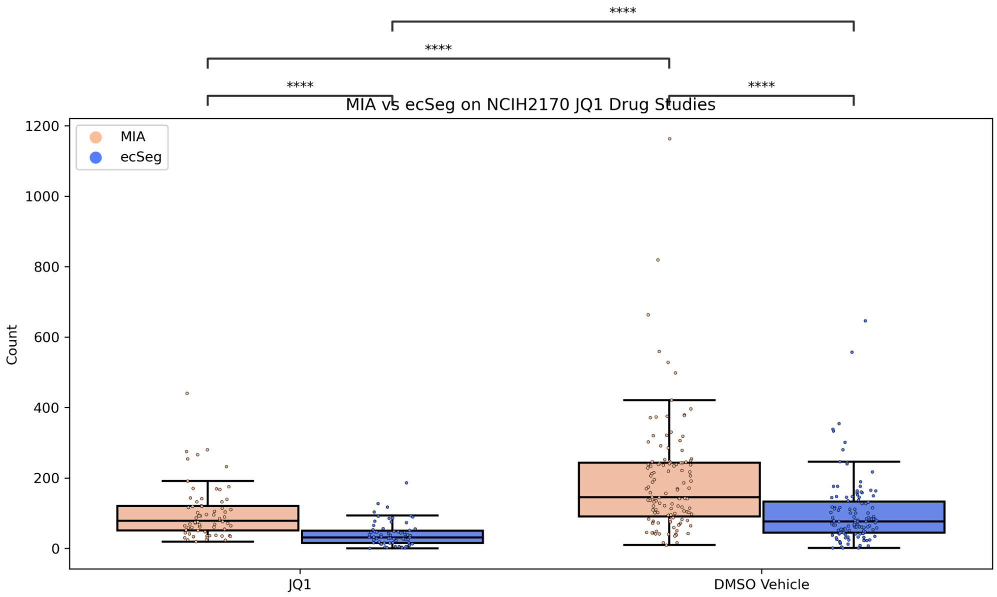

SI Figure 6 | Automated Counting of ecDNA changes in DMSO-only and JQ1-treated NCIH-2170 cells. Paired box plots show the ecDNA counts in NCIH2170 cells under control (DMSO-only) and JQ1-treated conditions, automatically quantified using MIA and ecSeg. Significant differences in ecDNA counts between control and JQ1-treated cells were observed across both prediction platforms (Mann-Whitney-Wilcoxon test, two-sided, with Bonferroni correction). For

JQ1-treated cells, predicted ecDNA counts differed significantly from the control using both MIA (p = 1.833e-6) and ecSeg (p = 6.086e-09).

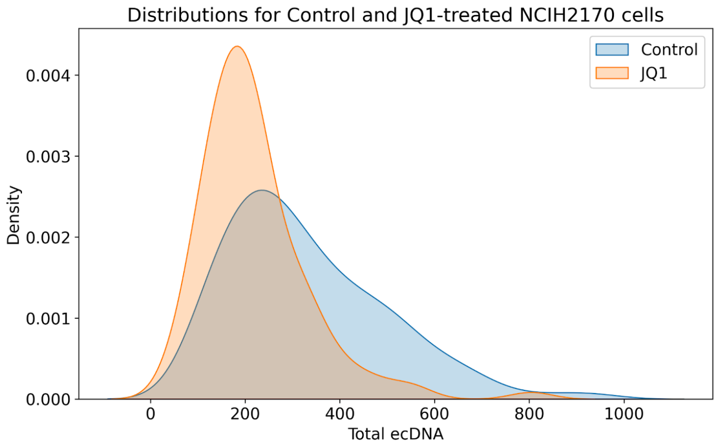

SI Figure 7 | Different distributions (total ecDNA) between Untreated and JQ1 treated NCIH2170 cells. Histogram displaying the distribution of total ecDNA counts per cell in untreated and JQ1-treated conditions. Statistical analysis demonstrates significant differences between the two distributions. The Kolmogorov-Smirnov (KS) test yielded a KS statistic of 0.381 and a P-value of 1.31×10^{-8}, while the Mann-Whitney U test resulted in a U statistic of 11464.5 and a P-value of 4.79×10^{-9}. These results indicate that JQ1 treatment significantly shifts the distribution of ecDNA values compared to the untreated population. The histogram highlights the variability in ecDNA levels and provides a visual representation of the observed differences.

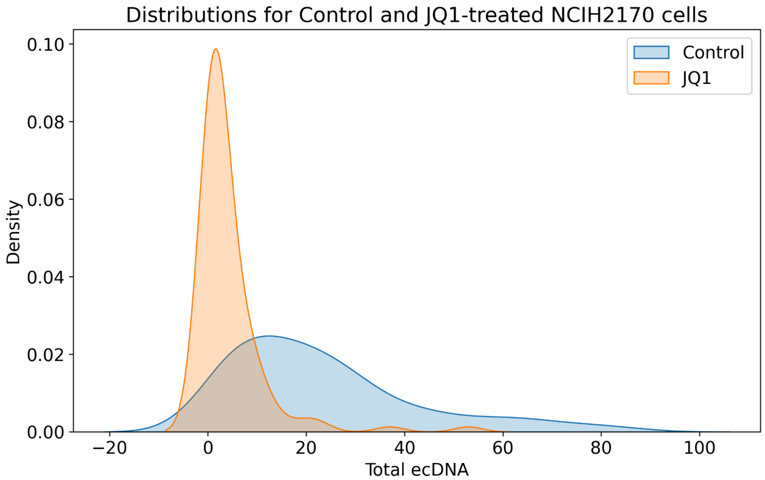

SI Figure 8 | Different distributions (MYC-only) between Untreated and JQ1 treated NCIH2170 cells. Histogram displaying the distribution of MYC-only localized ecDNA counts per cell in untreated and JQ1-treated conditions. Statistical analysis demonstrates significant differences between the two distributions. The Kolmogorov-Smirnov (KS) test yielded a KS statistic of 0.638 and a P-value of 1.47×10^{-24}, while the Mann-Whitney U test resulted in a U statistic of 14355.5 and a P-value of 3.69×10^{-27}. These results indicate that JQ1 treatment significantly shifts the distribution of MYC-only localized ecDNA counts compared to the untreated samples. The histogram highlights the variability in ecDNA levels and provides a visual representation of the observed differences.

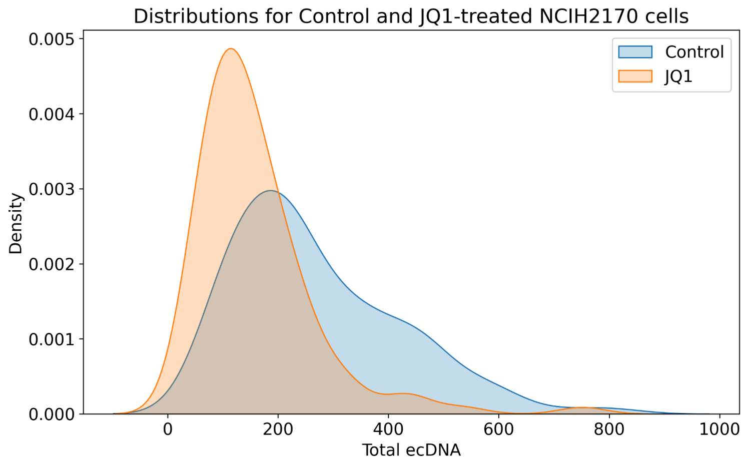

SI Figure 9 | Different distributions (MYC+ERBB2) between Untreated and JQ1 treated NCIH2170 cells. Histogram displaying the distribution of MYC+ERBB2 localized ecDNA counts per cell in untreated and JQ1-treated conditions. Statistical analysis demonstrates significant differences between the two distributions. The Kolmogorov-Smirnov (KS) test yielded a KS statistic of 0.397 and a P-value of 2.43×10^{-9}, while the Mann-Whitney U test resulted in a U statistic of 12070.0 and a P-value of 5.77×10^{-12}. These results indicate that JQ1 treatment significantly shifts the distribution of MYC+ERBB2 localized ecDNA counts compared to the untreated samples. The histogram highlights the variability in ecDNA levels and provides a visual representation of the observed differences.

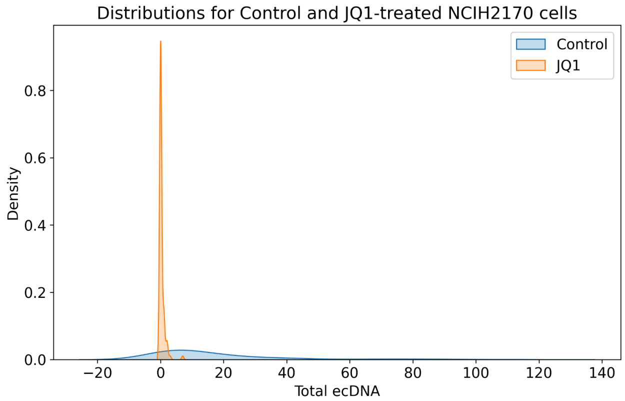

SI Figure 10 | Different distributions (ERBB2-only) between Untreated and JQ1 treated NCIH2170 cells. Histogram displaying the distribution of ERBB2-only localized ecDNA counts per cell in untreated and JQ1-treated conditions. Statistical analysis demonstrates significant differences between the two distributions. The Kolmogorov-Smirnov (KS) test yielded a KS statistic of 0.7946 and a P-value of 6.25×10^{-40}, while the Mann-Whitney U test resulted in a U statistic of 15337.5 and a P-value of 2.25×10^{-37}. These results indicate that JQ1 treatment significantly shifts the distribution of ERBB2-only localized counts compared to the untreated samples. The histogram highlights the variability in ecDNA levels and provides a visual representation of the observed differences.

Supplementary Tables

Metrics	NCIH 2170 3+		NCIH 2170 Control		NCIH 2170 JQ1	
	MIA	ecSEG	MIA	ecSEG	MIA	ecSEG
Precision	**0.311**	0.247	**0.260**	0.161	**0.301**	0.150
Recall	**0.567**	0.456	**0.464**	0.085	**0.304**	0.057
Dice	**0.400**	0.313	**0.332**	0.095	**0.293**	0.075
IoU	**0.252**	0.189	**0.201**	0.052	**0.175**	0.040
Matthew's	**0.418**	0.330	**0.345**	0.108	**0.297**	0.087
F1	**0.865**	0.806	**0.825**	0.436	**0.783**	0.364

Metrics	Ceiling Model		Full Model		CV Full Model	
	MIA	ecSEG	MIA	ecSEG	MIA	ecSEG
Precision	**0.322**	0.256	**0.256**	0.147	**0.263**	0.145
Recall	**0.595**	0.487	**0.356**	0.124	**0.337**	0.152
Dice	**0.417**	0.325	**0.286**	0.110	**0.278**	0.124
IoU	**0.265**	0.197	**0.172**	0.063	**0.167**	0.071
Matthew's	**0.437**	0.346	**0.295**	0.127	**0.287**	0.140
F1	**0.874**	0.819	**0.762**	0.415	**0.749**	0.435

SI Table 1 | Accuracies for predicting pixel-based locations of ecDNA. This table shows the pixel-based metrics for each trained model. Each pixel is considered an individual replicate within these metrics and compared to the ground truth maps to determine the accuracy. The MIA model outperforms ecSEG's model within each test data set for the corresponding training data for all available metrics.

**Model**	Actual MYC	Pred MYC	Actual ERBB2	Pred ERBB2	Actual MYC+ERBB2	Pred MYC+ERBB2	Total ecDNA
2170 JQ1	3.369	15.477	0.338	1.293	165.954	147.662	165.379
2170 Control	23.053	35.840	17.680	21.227	284.133	261.187	326.675

SI Table 2 | Accuracy metric for detecting probes on MIA-predicted ecDNAs. This table summarizes the results from our automated probe counting script which was tested on two subsets of our NCIH2170 dataset: JQ1 and control. In each of our three probe categories (MYC, ERBB2, and MYC+ERBB2), our script gave counts closer to ground truth for the control subset compared to JQ1. Furthermore, our script is best at identifying ecDNAs with both MYC and ERBB2, followed by ecDNAs with just ERBB2, and has the most difference from ground truth for ecDNAs with just MYC.

<u>HOW TO Documentation</u>
MIA & Masking-Ranking Images

Masking

1. Download ImageJ/FIJI (<u>Download Link</u>)

2. Open FIJI (click the ImageJ-win64.exe)

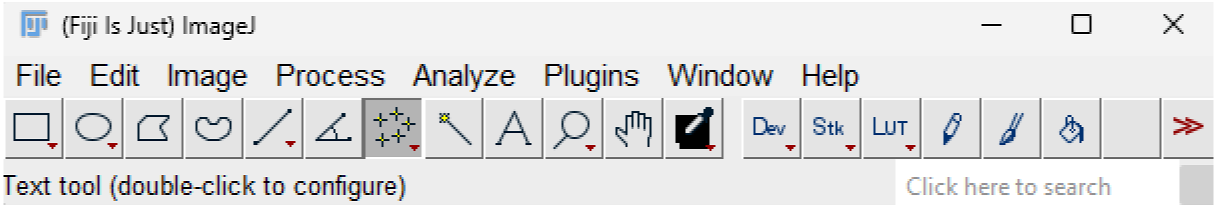

- The **File Edit Image…** will be in the top right of the screen if on MAC

3. Once the tool bar is open find, either find the folder you wish to process or click **File**, then **Open…**, and navigate to the folder of interest
   - Notice that sometimes the organization of images can change based on if you open it through the second method and will not follow the same organization if you click **Open Next (Ctrl+Shift+O)**
   - Some images are corrupted and will not have annotations or will give you an opening prompt with many options such as open with or without hyper stack. *Make Notes of these images in ranking and give them a 0*

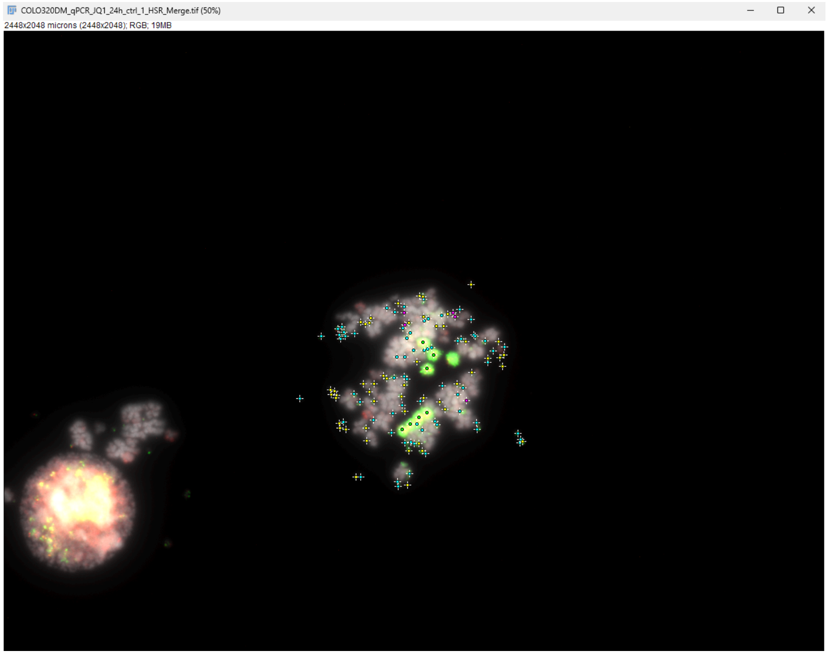

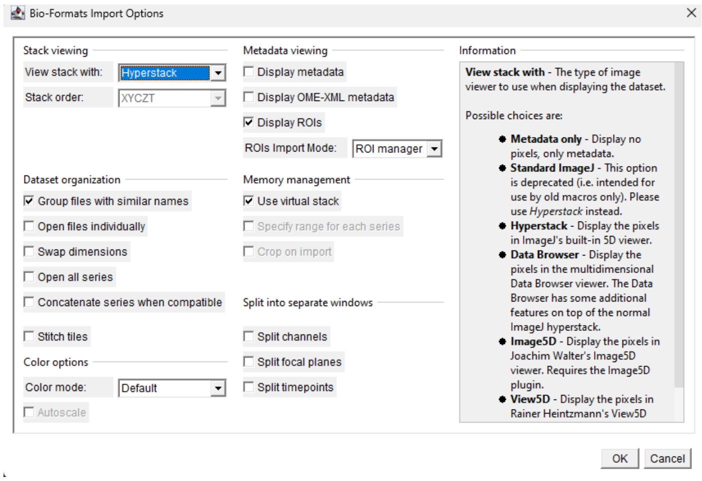

4. With the full image open with annotations, we will open 3 extra windows to Point tool, Brightness/Contrast, and ROI Manager. Follow the steps to find each below

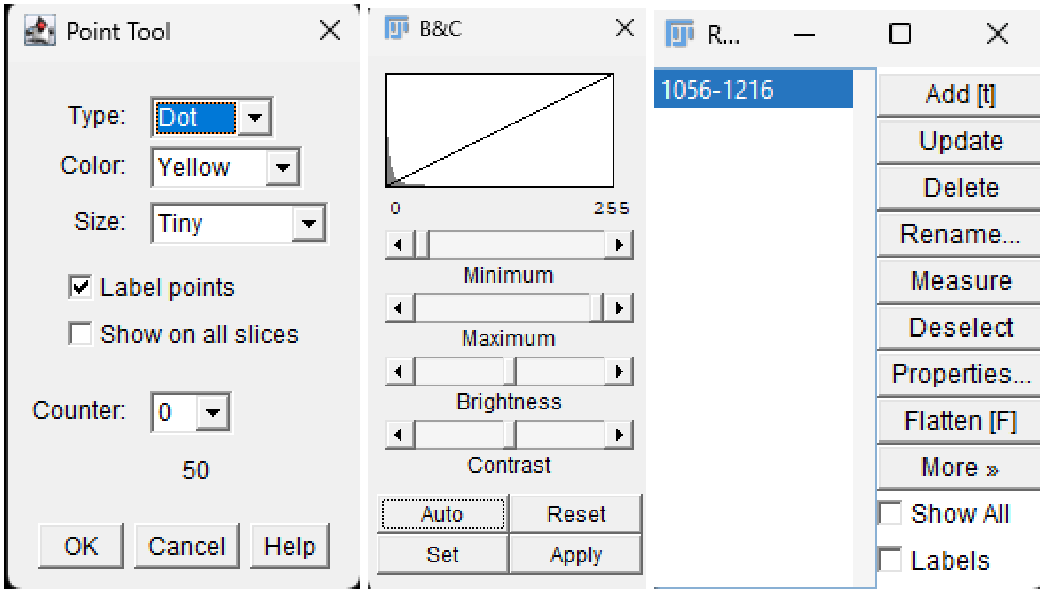

- Point tool: **Double Click the** 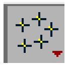 **Icon**

- Contrast/Brightness: **Image, Adjust, Brightness/Contrast…**
  - Only slide the bar above Maximum and click reset before saving every time

- ROI Manager: **Analyze, Tools, ROI Manager…**

5. Click on the open image then click **Add[t]**, a couple numbers will pop up on the tab which will correspond to the save location of the annotations
6. To separate the images into the different Channels (DAPI, Probe1, Probe2…) click **Image, Color, Split Channels,** on the pop up **Dont Save.** A (red),(green), and(blue) window will pop up.
- This step can be skipped if already only in the DAPI (grayscale) channel

7. If the blue window is on the top, double click the **Show All** check box and the annotations should pop up on the DAPI (blue) channel image
8. Increase the Maximum bar on the B&C window to almost full, to the point where the image looks like the following to highlight potential debris(if you do not have the

annotations in ROI Manager and try this it will result in a square of the annotations to not change):

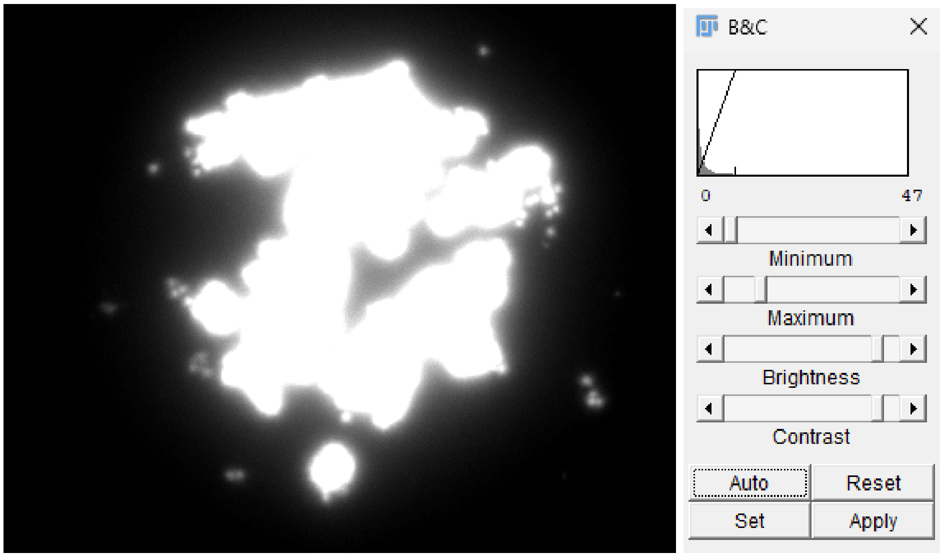

9. Click on the **Freehand Selection** 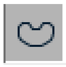 **Icon** and highlight around the annotations or the single nuclei full splash radius if novel images

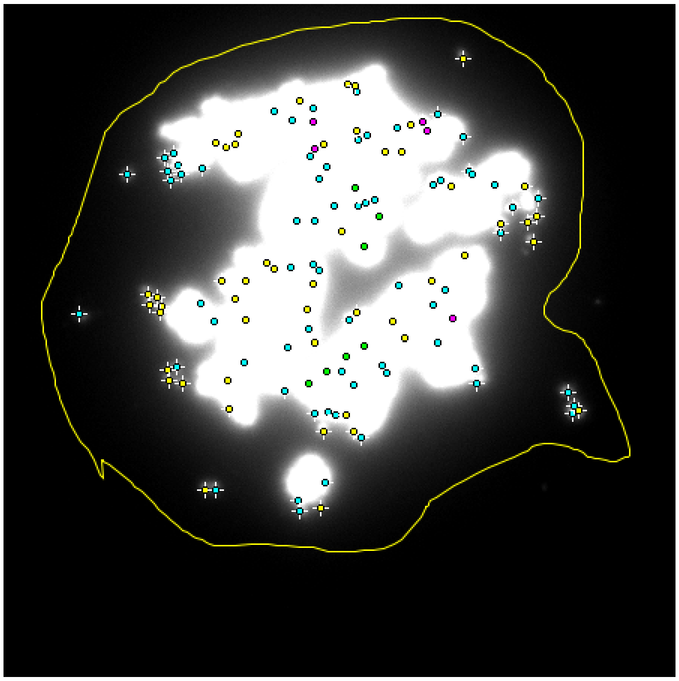

10. Right click on the window inside the outlined ROI, then click **Create Mask**

● If the image is just be cropped reducing it to just the ROI, click on **Edit, Clear OutsideSave** then **File, Save**

11. To save the resulting mask you can either save as a direct image or oversave the original image where you don’t need to rewrite the image name. To do the oversave, make sure a copy of the images exist in an auxiliary folder. Click **File, Save as, Tiff…,** then Click the corresponding image you masked and **Save**

Ranking

Each should be ranked on 2 major factors, Image Resolution/Quality and Annotation Quality. Overall resolution can be determined fairly confidently using the merged images but annotations should be reviewed in the DAPI (Grayscale) channel. Below is a table of examples of each rank 0-4 and description on what to look for. Ranking and masking can be done either separately or during the same time.

1. Make an initial judgment based on the image resolution as a whole. Such as if it is super blurry or has strange/distant annotations
2. Follow Steps 4-7 in the masking steps
3. Rapidly click the **Show All** check box and see if all the annotations line up approximately with the ecDNA dots and if there are annotations on top of chromosomes or very far from the center of the nuclei
4. Judge the image based off the ranking guidelines below and update the rank in the shared Google Sheets(link)

a. *If making edits or improvements*, click the **point tool** then click to highlight the numbers in the **ROI Manager.** Hover over annotation locations you wish to remove (Ctrl+click[just hover and click on MAC]) or add (click).

b. Re-open the original merged image if you closed it out then double click the **show all** check box in the **ROI Manager.** This will update the annotations on the image to the set with the augmentations

c. Save the image with improved annotations via **File, Save**

Image Ranking Guidelines

Ranks should be made on what the images current condition is

Guideline for ranking with examples:

0	Major Counting Errors (only HSRs,<br>ecDNA on top of the chromosomes of most of DAPI ecDNA are missing),<br>very problematic image location, very poor resolution<br>(should be considered for removal)

	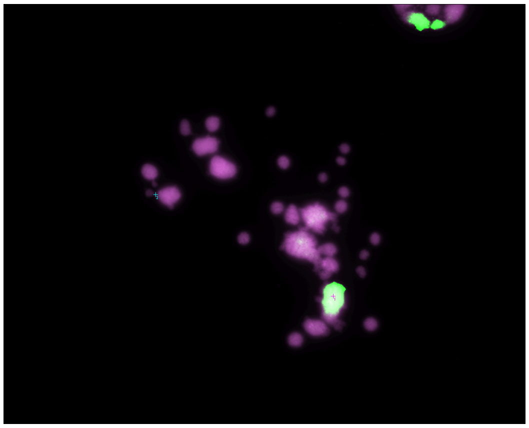 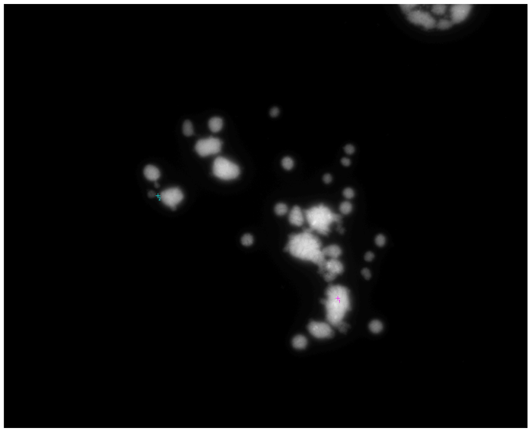 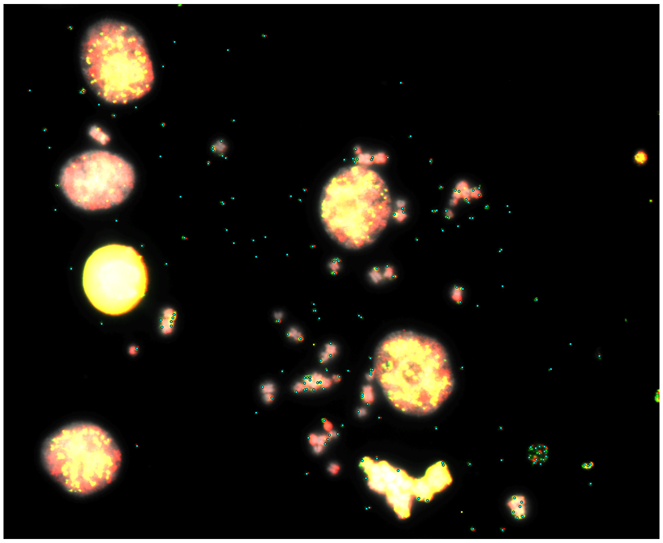
1	Poor nuclei location/splash/quality,<br>poor counting quality, hard to visualize/non-circularized ecDNA in DAPI<br>(should be considered for only human counting or reviewed closely if model counted)<br>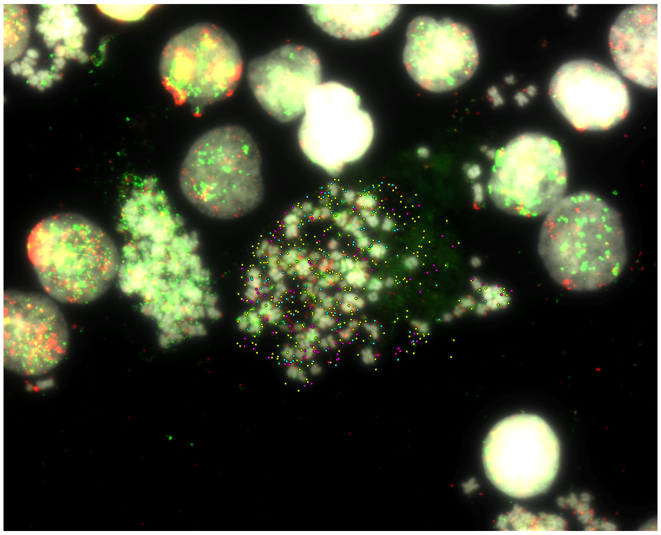

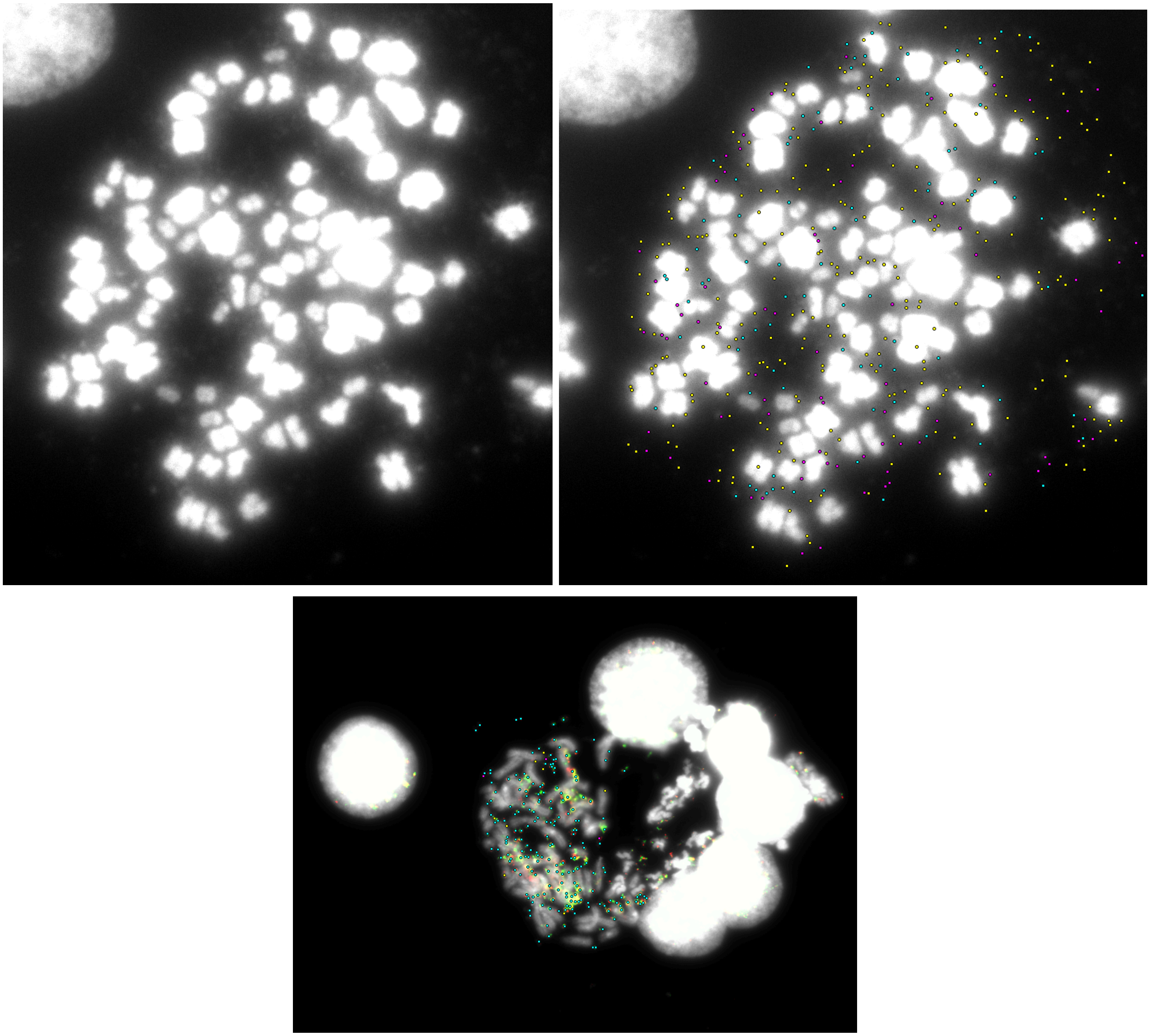

<table>
<tr><td></td><td>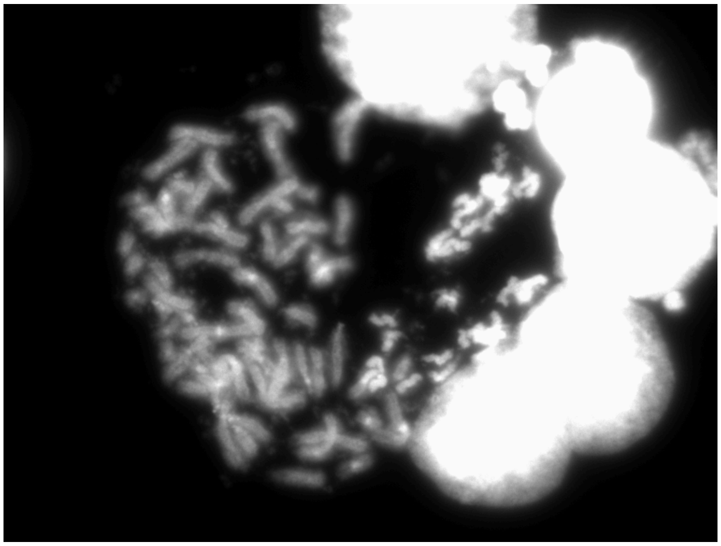 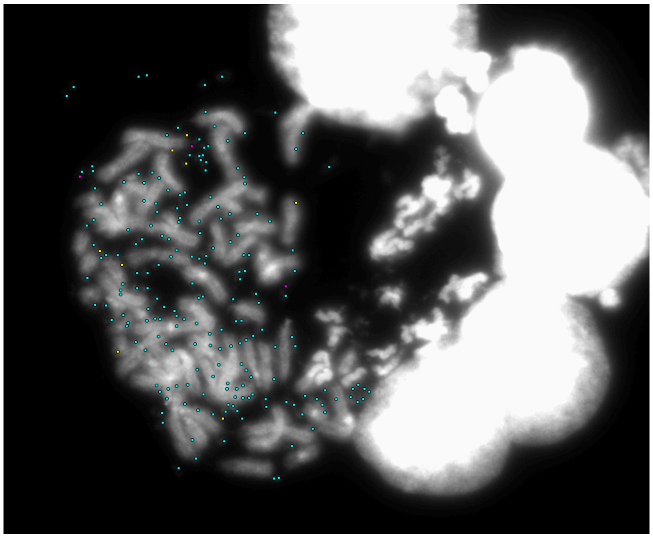</td></tr>
<tr><td>2</td><td>Arbitrary nuclei splash size, lots of artifacts within nuclei splash zone,
some systematic counting errors (counting of ecDNA is slightly off center due to little probe overlay),
images with blur field around chromosomes or separation cannot be seen
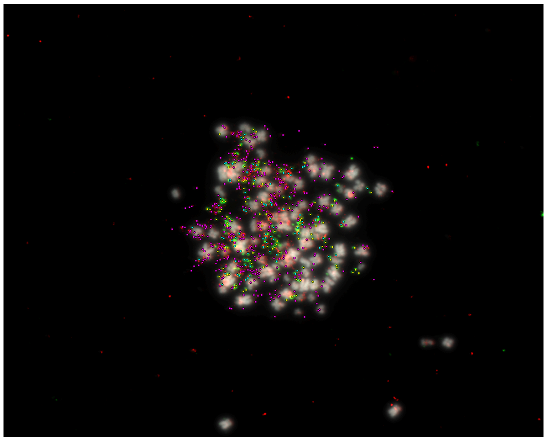
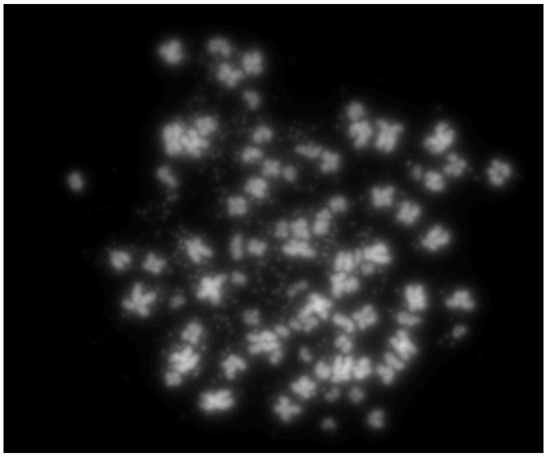 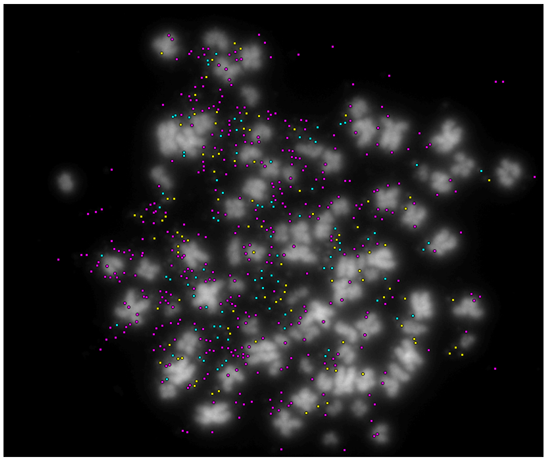</td></tr>
</table>

	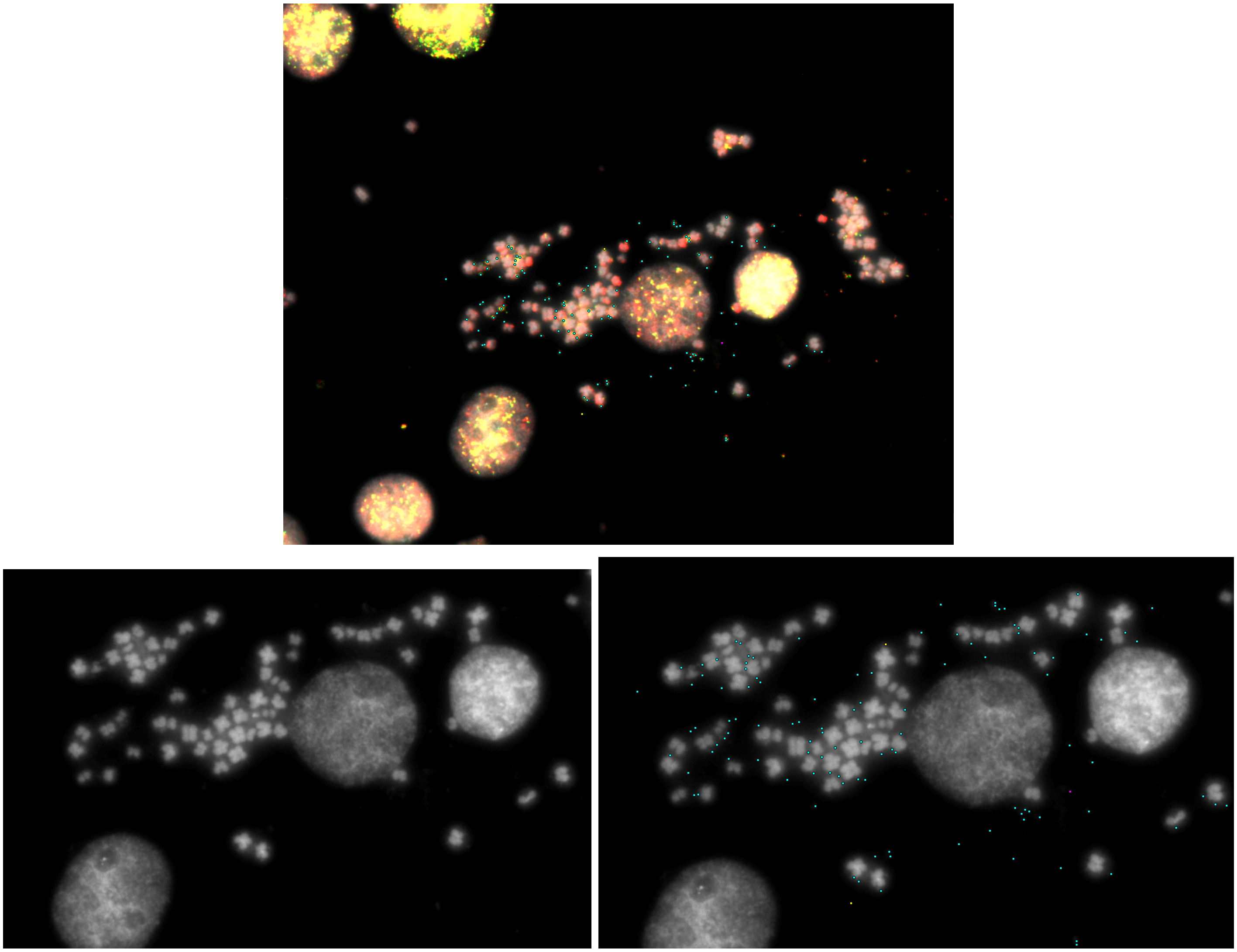
3	Relatively small drift, low count error some adjacent objects/nuclei, decent image resolution

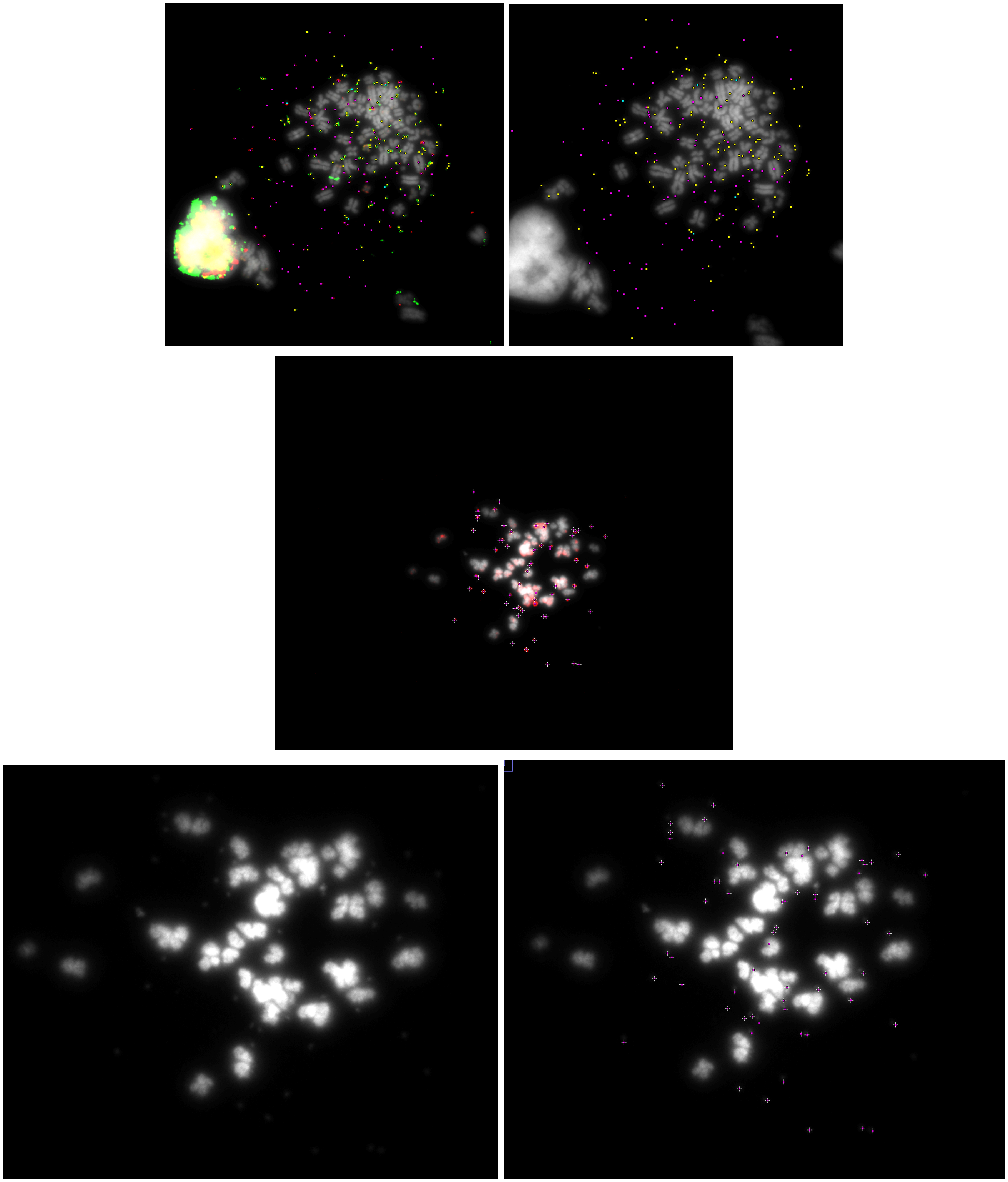

4	High count confidence, clear/small splash zone, clear resolution<br>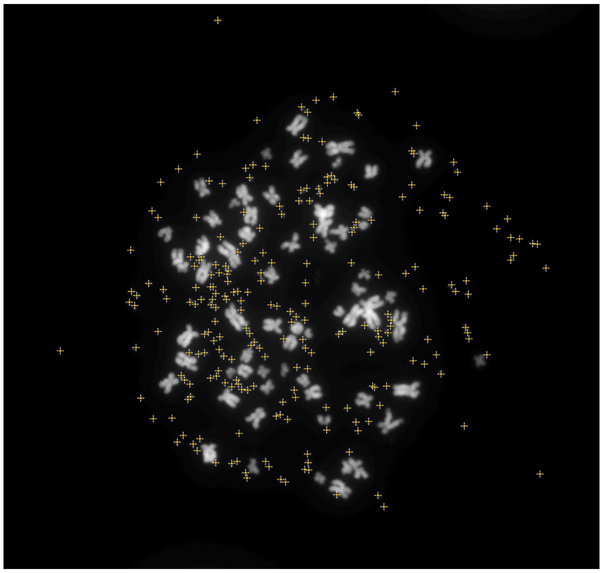<br>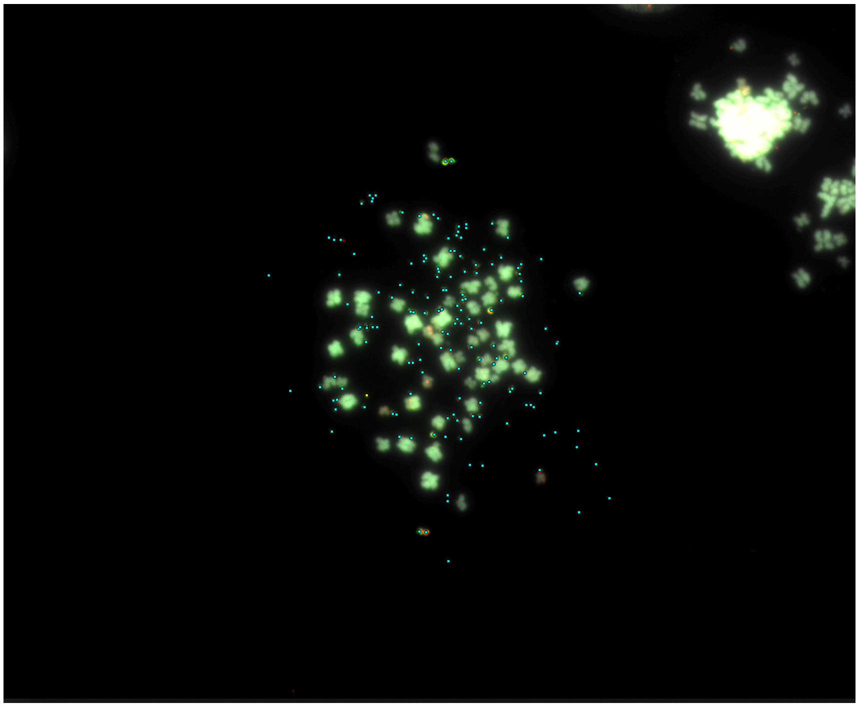

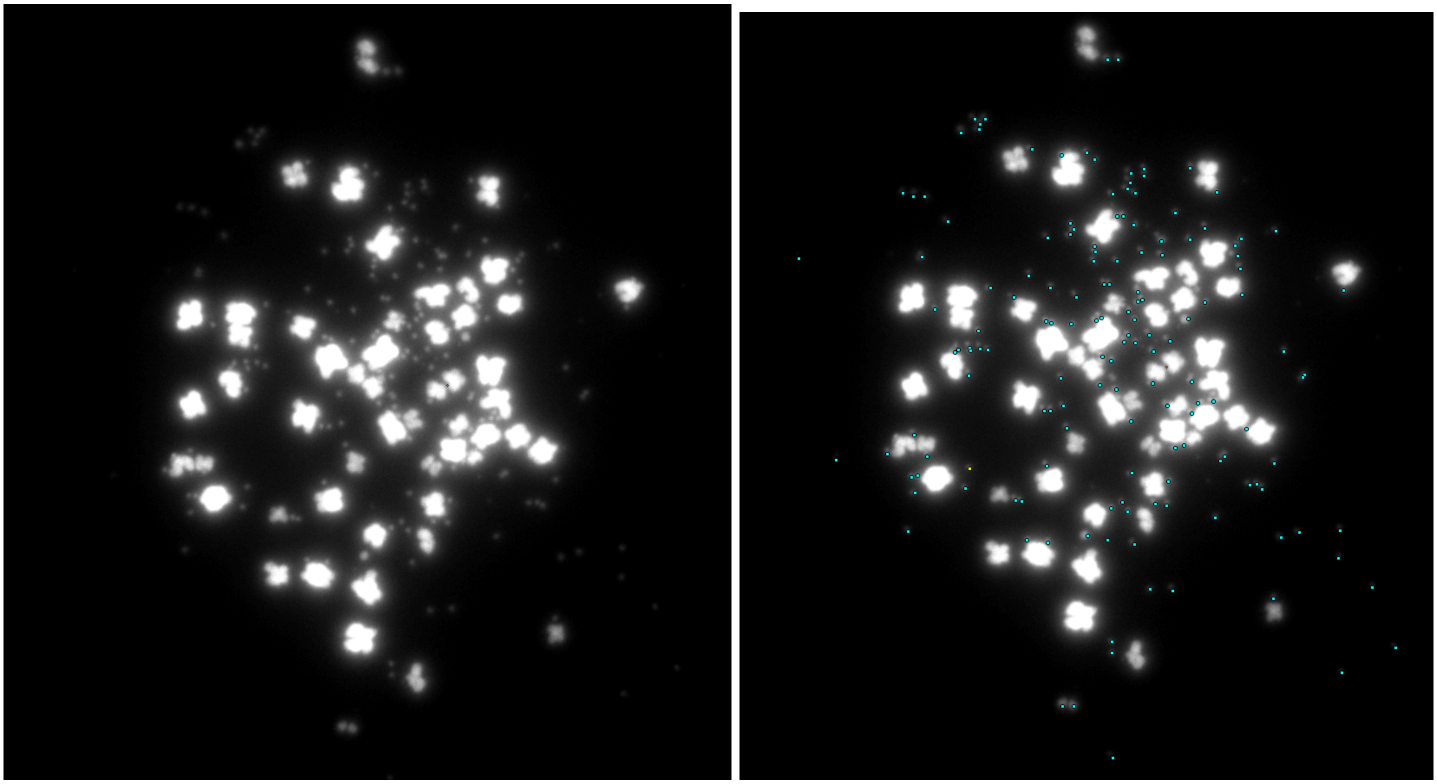

Ranking system:

0	Major Counting Errors (only HSRs or most are missing),<br>Problematic image location, Poor resolution (should be considered for removal)
1	Poor nuclei location/splash/quality, poor counting quality, (should be reviewed)
2	Arbitrary nuclei splash size, artifacts within nuclei splash zone, some systematic counting errors
3	Relatively small drift and some adjacent objects/nuclei, decent image resolution
4	High count confidence, clear/small splash zone, clear resolution

Example with ecDNA w/o probe being skipped, slight blur rank:2/3

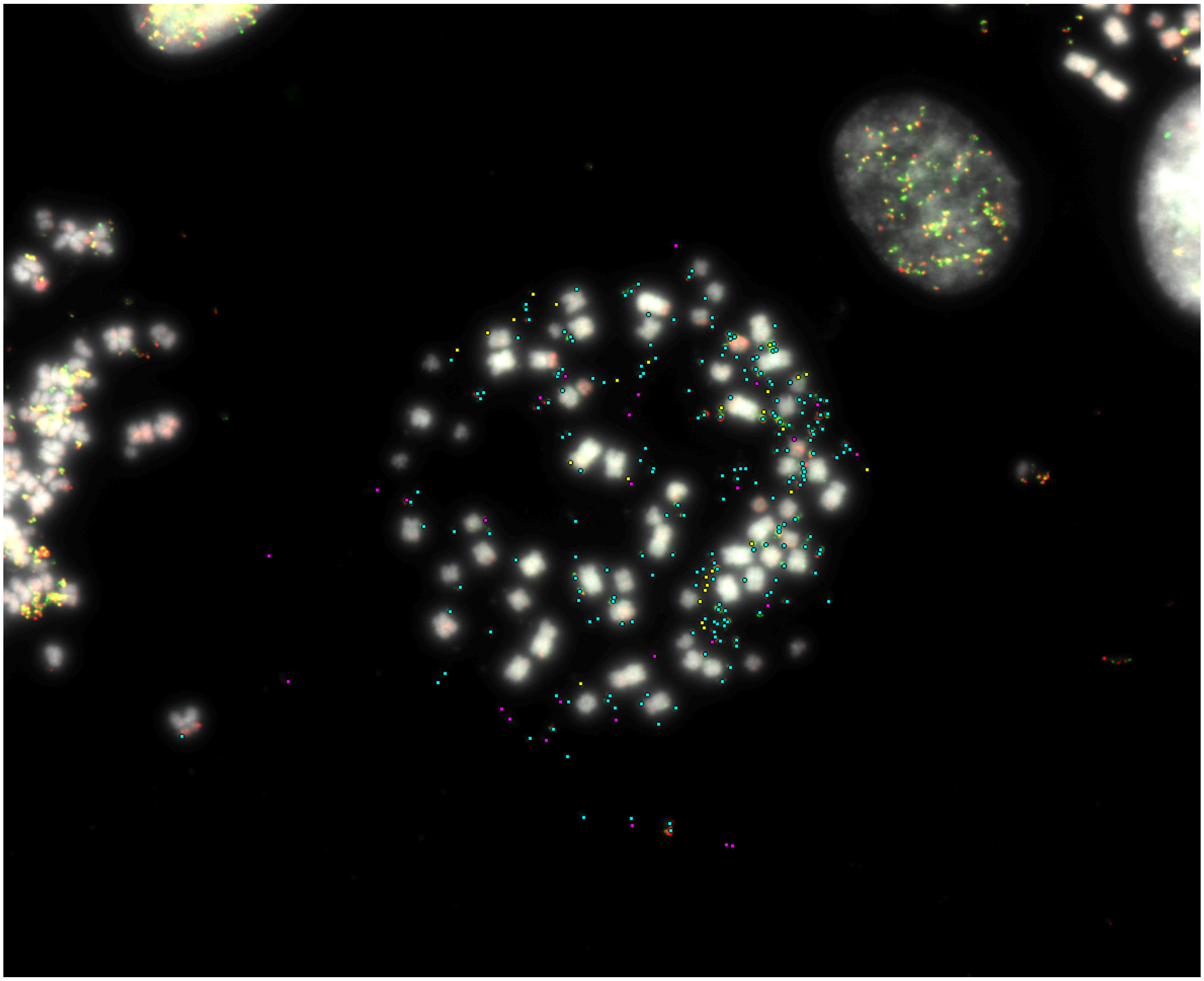

Running MIA

Most of the needed information for running and modifying MIA can be found either on the github (link), paper (link), or manual (link). This guide is an additional help if needed.

Activation

1. Download the MIA environment and follow instructions from this link (Download and Setup Link)
   - Depending on if you are downloading MIA to a cloud OS or local will change some operations that can be used, for example when using MIA in Longleaf automatic weighing for learning rate, multiple GPU parallelization, nor contour detection during training can be used.
2. Once the environment is installed follow the list of commands below to launch MIA
   a. Module purge
   b. Module load anaconda
      i. (customize to your own path) export LD_LIBRARY_PATH=/nas/longleaf/home/koandgo/.conda/envs/mia_environment/lib:$LD_LIBRARY_PATH
   c. Conda activate mia_envrionment
   d. Mianalyzer
   - Skip straight to C if you can directly open an anaconda terminal for personal use
   - Substep b is only needed for MIA on Longleaf since certain libraries or versions are not directly accessible
3. In the MIA UI load a training data set with a folder named Segmentation_labels(contained with same # and name of images as npz files)
4. A model can either be uniquely trained or a preexisting one can be loaded
   a. To load a model simply click the load model button and find the .h5 file for the model to load
5. The training parameters can be selected from the train model pop-up. The default conditions were used for most models generated
6. Once the conditions are set, click the start training button on the pop-up and an additional window will be launched showing the loss and stats in real time.
   - If you decide to stop the models training early (either due to memory concerns or loss plateaus, stop the training and the most recently completed epoch and its weights can be saved manually then re-launched again from that position.

7. Once a model is finished, its accuracy can be determined using predictions and the test image folder
   a. Click post processing and click the check box for contour separation on prediction and choose sizing parameters
      1. Used 3 as min contour size and 3 as min contour distance

8. With the Test image folder highlighted, click the predict all button and wait for results
   a. Progress can be visualized in the bottom right corner
9. Click the results button below post processing and a csv of results can be saved along with the masks for each of the images which can then be analyzed externally using python
   - This can take a long processing time to complete based on the size of the csv that will be generated and the processing power available
